# Living and working through health uncertainty: An analytic autoethnography of identity, strain, and future-making

**DOI:** 10.1016/j.qrmh.2026.100049

**Published:** 2026-09-01

**Authors:** Mehmed Zahid Çögenli

**Affiliations:** Department of Occupational Health and Safety, Faculty of Health Sciences, Uşak University, Uşak, Türkiye

**Keywords:** Prolonged health uncertainty, Analytic autoethnography, Biographical disruption, Occupational health psychology, Professional identity, Work and health

## Abstract

**Background:**

Prolonged health uncertainty extends beyond diagnostic processes and clinical encounters into everyday life, reshaping work, relationships, self-understanding, and the capacity to imagine a stable future. Yet the occupational and relational burden of living under uncertainty often remains hidden when outward functioning is preserved.

**Method:**

This analytic autoethnography examined how prolonged health uncertainty was lived and negotiated across work, family life, and selfhood. The analysis drew on a curated archive of self-authored materials, including contemporaneous written reflections, retrospective accounts, and selected self-narrative documents. Materials were interpreted thematically through the lens of biographical disruption.

**Results:**

Four interrelated themes were identified: 1) health uncertainty beyond the clinic, 2) carrying competence under strain, 3) silence, disclosure, and institutional vulnerability, and 4) rebuilding a livable future. The findings show that uncertainty became embedded in the ordinary structure of life rather than remaining a purely medical issue. Continued functioning at work depended on self-monitoring, emotional containment, selective silence, and repeated efforts to preserve a coherent professional self. Uncertainty remained unresolved, but was incorporated into a more fragile, still livable continuity.

**Conclusions:**

Prolonged health uncertainty should be understood as both a medical problem awaiting clarification and a disruptive occupational and relational condition. This article highlights the hidden labor involved in remaining apparently functional while living and working through uncertainty.

## Introduction

On some mornings, deciding how to move through the day without drawing attention to what remained unexplained came before teaching, writing, or replying to emails. Health uncertainty emerged gradually, rather than as a single dramatic event. It accumulated quietly—through symptoms that resisted straightforward everyday explanations, through questions that outpaced answers, and through the growing realization that work could continue outwardly even when certainty had already begun to collapse inwardly. In the space between visible competence and private disruption, ordinary academic life became a site where bodily doubt, emotional restraint, and professional responsibility had to be managed at the same time.

I write from within this experience as an academic who remained at work while living with prolonged health uncertainty. I continued teaching, supervising, traveling, producing, and trying to remain reliable to others, even as uncertainty began to reshape how I inhabited my body, my work, and my future. I was dealing with symptoms and with the possibility of diagnosis; I was also dealing with what uncertainty did to my sense of self, to my relationships, and to the practical labor of remaining functional. The struggle was therefore never only medical. It unfolded across work routines, family responsibilities, emotional self-management, and the constant effort to preserve a coherent professional identity under strain.

This article is framed through the concept of biographical disruption, which helps illuminate how health-related uncertainty can unsettle the taken-for-granted continuity of everyday life. In my case, disruption emerged through prolonged uncertainty itself—the not-yet-known, the not-yet-confirmed, and the not-yet-resolved condition that gradually entered work, family life, and selfhood. Biographical disruption is useful here because it allows health uncertainty to be understood as both a private psychological difficulty and a force that reorganizes time, meaning, relationships, and expectations about who one is able to be. It also opens space to examine the practical and emotional work required to keep living and working while certainty remains out of reach.

Accordingly, this article offers an analytic autoethnography of how prolonged health uncertainty became embedded in occupational life and everyday identity. Drawing on contemporaneous written reflections, retrospective accounts, and selected self-narrative materials, I examine how uncertainty was lived as a health concern and as a struggle over competence, disclosure, responsibility, and future-making. Rather than presenting a technical account of diagnosis, I focus on how uncertainty was experienced, interpreted, and negotiated across the overlapping domains of work, family life, and selfhood. In doing so, I argue that prolonged health uncertainty can function as a deeply disruptive social and occupational condition, even before clinical certainty is achieved.

Although uncertainty is often discussed in relation to diagnosis, treatment, and communication, its lived effects are rarely confined to clinical settings. A substantial body of work has shown that chronic or unresolved health problems can disrupt bodily routines, identity, social roles, and assumptions about continuity in everyday life (Bury, 1982, Charmaz, 1991, Cluley et al., 2021, Estecha Querol et al., 2020, Frank, 1995). Yet the experience of uncertainty extends beyond the moment of diagnosis and medical decision making. For many individuals, uncertainty becomes an ongoing condition of living that stretches across work obligations, family roles, emotional regulation, and practical decisions about how to move through ordinary time. In this sense, health uncertainty extends beyond the epistemic; it is also organizational, relational, and biographical. It unsettles both what a person knows and how a person works, plans, performs competence, and imagines a future self.

This matters especially in contexts where people are expected to remain reliable, productive, and professionally coherent while privately managing instability. Research on chronic illness has long emphasized the labor involved in sustaining everyday life under altered bodily conditions, including the practical, emotional, and relational work required to maintain routines and preserve social participation (Corbin and Strauss, 1985, Corbin and Strauss, 1988). At the same time, narrative and interpretive traditions have shown that illness and health disruption are endured and made meaningful through ongoing acts of interpretation, reinterpretation, and self-revision (Frank, 1995, Hydén, 1997). However, much of this literature has focused either on illness identity broadly or on clinical uncertainty as a patient-care issue. Less attention has been paid to what prolonged health uncertainty does to occupational life when a person is still expected to function, care for others, and uphold a credible professional self in the absence of stable resolution.

This gap is particularly important for understanding the hidden burden of remaining “apparently functional.” People may continue to teach, manage, write, parent, commute, and meet expectations while uncertainty quietly reorganizes the conditions under which these activities are carried out. The issue, then, extends beyond whether one is ill to how one continues to inhabit work and responsibility when certainty has receded. Here, biographical disruption offers a powerful starting point because it highlights how health-related disturbance can fracture the taken-for-granted structure of everyday life (Bury, 1982). But in prolonged conditions of uncertainty, disruption may not appear as a single rupture. It may instead unfold cumulatively, through repeated adjustments, strategic silences, altered self-observation, and the slow erosion of confidence in one’s own continuity. Read this way, uncertainty becomes both an interruption and an ongoing demand to reorganize selfhood while life continues.

This article takes up that problem through analytic autoethnography. Autoethnography is particularly suited to experiences that are difficult to capture through detached description because it allows the researcher to examine lived experience from within while also subjecting that experience to analytic interpretation (Anderson, 2006). In health-related contexts, such work can illuminate forms of burden that are normalized, hidden, or difficult to articulate in conventional academic language. Analytic autoethnography is especially valuable when the experience examined here is neither reducible to a clinical timeline nor fully visible to others, but instead unfolds across mundane routines, relational obligations, and the private effort to remain socially legible. For that reason, this article approaches health uncertainty as a lived condition rather than a bounded medical event. In my experience, it gradually became embedded in work, family life, and self-understanding, with uneven and consequential effects.

My aim is therefore to examine how prolonged health uncertainty became woven into the everyday work of being a professional, a family member, and a person trying to preserve continuity under strain, rather than to provide a technical account of disease or document diagnosis as a singular endpoint. In doing so, I extend the discussion of biographical disruption by showing how disruption may persist through both illness itself and the prolonged absence of clarity. The argument advanced here is that health uncertainty can function as a disruptive occupational and relational condition in its own right—one that shapes competence, silence, emotional containment, responsibility, and future-making even before certainty is reached.

The article proceeds by examining four interrelated dimensions of this experience: 1) the movement of uncertainty beyond the clinic and into everyday life, 2) the effort to carry professional competence under strain, 3) the management of silence, disclosure, and institutional vulnerability, and 4) the gradual rebuilding of a livable future. Together, these themes show that neither a narrow medical lens nor a purely psychological one adequately captures the experience at stake. Rather, it emerges at the intersection of embodiment, work, relational responsibility, and biography. What follows, then, is an account of how health uncertainty was lived, interpreted, and negotiated as an everyday struggle to remain intact while the grounds of ordinary continuity were shifting.

## Method

### Analytic orientation

I used analytic autoethnography, an approach that combines sustained attention to lived experience with explicit analytic interpretation (Anderson, 2006). Autoethnography is broadly understood as a method that connects personal experience with wider cultural, relational, and institutional contexts, using the researcher’s situated life as both a source of narrative and a site of analysis (Ellis et al., 2011; Adams et al., 2022). In this tradition, good autoethnographic writing requires more than self-disclosure; it requires reflexive attention to craft, analysis, ethics, and the movement between lived experience and cultural meaning (Adams & Herrmann, 2023). Within academic and doctoral contexts in particular, autoethnography has also been used to illuminate experiences of strain, identity work, and wellbeing that are otherwise difficult to articulate (Pretorius & Cutri, 2019).

Autoethnography was appropriate because the phenomenon examined here—prolonged health uncertainty entering work, family life, and self-understanding—was difficult to observe externally or capture adequately through detached description alone. What was at issue was both what happened and how ongoing uncertainty was lived, managed, interpreted, and incorporated into everyday routines and responsibilities over time.

I adopted an analytic, rather than purely evocative orientation, because the aim of the article is to examine, rather than simply narrate, personal experience as a site through which broader questions about health disruption, occupational functioning, silence, responsibility, and future-making can be understood. In this sense, I treat personal experience as a source of situated knowledge requiring reflexive engagement and conceptual interpretation, rather than as self-expression alone.

### Analytical lens: biographical disruption

Biographical disruption was used as the primary sensitizing lens for the analysis. The concept refers to the way illness can disturb the taken-for-granted structures through which people understand their bodies, identities, relationships, routines, and imagined futures (Bury, 1982). Its value here lies in its capacity to show that health-related difficulty can be understood as a biological or clinical event and a disruption of ordinary continuity. Illness, or the possibility of illness, can unsettle aspects of life that previously felt stable, including the reliability of the body, the coherence of the self, the predictability of work, and the ability to imagine a future on familiar terms.

Here, biographical disruption serves as an interpretive lens rather than a rigid coding frame for examining how prolonged uncertainty reorganized everyday life before clinical certainty was fully available. This distinction is important. The disruption analyzed here began before a settled diagnosis or a clearly bounded illness identity had formed. It emerged through the ongoing absence of clarity, the need to keep functioning despite uncertainty, and the gradual reworking of work, family life, disclosure, responsibility, and future-making. In this sense, this autoethnography extends biographical disruption scholarship by showing that uncertainty itself, as well as diagnosis or established illness identity, can become biographically disruptive.

### Researcher position and context

I am both the author of this manuscript and the person whose experience forms the basis of the analysis. At the time reflected in these materials, I was working within academic life while simultaneously managing prolonged health-related uncertainty that affected my bodily awareness, work routines, family responsibilities, emotional regulation, and sense of continuity. This dual position—as experiencing subject and analytic interpreter—was treated as a methodological condition to be made explicit rather than a limitation to be removed.

Because the experience unfolded within ongoing professional and relational obligations, the analysis attends to health-related meanings and to how uncertainty shaped ordinary efforts to remain competent, dependable, and socially recognizable. Rather than claiming distance from the experience, I sought to develop analytic clarity through reflexive examination of how the experience was recorded, revisited, and interpreted across time.

### Data sources

My analysis drew on a curated set of self-authored materials produced across the broader period of health uncertainty. The core of the archive consisted of approximately 1150 lines of contemporaneous reflexive writing in its raw form, produced as part of ongoing efforts to make sense of emerging health-related concerns, their implications for professional life, and their impact on future orientation. Alongside these records, the archive included retrospective reflective accounts and selected self-narrative documents concerning work, family life, and personal meaning-making. Together, the materials captured evolving interpretations, emotional responses, and identity negotiations across time, forming an archive of lived interpretation, rather than a single retrospective narrative reconstructed after the fact.

The contemporaneous materials were especially important because they documented uncertainty as it unfolded. They captured moments in which meanings had not yet stabilized and in which work, strain, responsibility, and self-observation were still being negotiated in real time. Retrospective reflections and selected self-narrative documents complemented those earlier records by placing them in temporal perspective and showing how some experiences were later reorganized, reinterpreted, or made narratable.

Because the article focuses on selected aspects of the broader archive, the materials used here were analytically delimited. Materials were included when they spoke directly to the ways health uncertainty entered everyday functioning, occupational life, family responsibility, emotional containment, silence or disclosure, and the effort to maintain continuity under strain. Materials that were primarily technical, medically descriptive, or peripheral to these concerns were not foregrounded in the present analysis.

### Data selection and analytic process

Analysis proceeded in several stages. First, I reviewed the broader archive repeatedly in order to identify episodes in which health uncertainty became visible as both a bodily concern and a disruption to everyday continuity. At this stage, I was attentive to moments involving altered work functioning, strategic silence, shifts in self-monitoring, heightened responsibility, disrupted future planning, and efforts to preserve competence under strain.

Second, I developed analytic memos for selected excerpts and episodes. These memos focused on what each passage revealed about the lived organization of uncertainty, including how it shaped action, what forms of emotional or practical labor it required, what it threatened, and how it reorganized my relation to work, family life, and selfhood. The purpose of memoing was to move beyond description and to ask what the material was showing analytically.

Third, I compared excerpts across time and context in order to identify recurring patterns rather than isolated moments. This comparative reading helped distinguish transient reactions from more durable interpretive structures. Through this process, initial observations were gradually clustered into broader thematic formations. Themes were not treated as pre-existing categories waiting to be extracted; rather, they were developed iteratively through movement between the archive, memo-writing, and conceptual reflection.

This process resulted in four interrelated themes: 1) health uncertainty beyond the clinic, 2) carrying competence under strain, 3) silence, disclosure, and institutional vulnerability, and 4) rebuilding a livable future. These themes were selected because they captured recurrent content as well as the underlying structure of the experience as it unfolded across multiple domains of life.

### Reflexivity and an interpretive approach

Given the self-implicated nature of the material, reflexivity was central throughout the analysis. Rather than assuming that proximity to the experience guaranteed transparency, I treated my own interpretations as shaped by time, emotional intensity, later understanding, and the practical need to make difficult experiences narratable. For this reason, I moved repeatedly between contemporaneous records and later reflections, asking what had happened and how different forms of writing preserved, condensed, displaced, or reorganized what had been lived.

Interpretation was guided primarily by the concept of biographical disruption (Bury, 1982), which provided a way of examining how prolonged health uncertainty unsettled continuity in work, family life, and self-understanding. This concept functioned as an interpretive lens that helped illuminate how uncertainty was carried, normalized, concealed, and gradually woven into the labor of everyday life, without imposing a rigid coding frame.

### Translation and privacy note

Excerpts originally written in Turkish were translated by the author and lightly edited for clarity, flow, and the protection of privacy. These edits preserved the substantive meaning of the original reflections.

### Ethical considerations

This autoethnography draws exclusively on my self-authored materials and required no recruitment of external participants. Because I am both the author and the participant in this autoethnographic work, selecting, interpreting, and sharing my stories constitutes my informed consent. However, the self-focused nature of this work still entails ethical responsibility toward others who appear indirectly in the account.

For this reason, potentially identifying details concerning third parties were minimized, omitted, or described at a level of abstraction intended to protect privacy and relational confidentiality. The analysis avoids adjudicating the motives, identities, or conduct of other individuals. Instead, it focuses on lived process, meaning, vulnerability, and the relational and institutional conditions through which health uncertainty was experienced. This approach reflects a relational understanding of autoethnographic ethics, in which the author’s right to tell a self-narrative is balanced against the responsibility to avoid unnecessary exposure or harm to others (Bochner, 2007; Edwards, 2021).

## Living through uncertainty

The analysis developed across four interrelated themes. Together, these themes show that prolonged health uncertainty was more than a purely medical issue or private emotional burden; it entered the practical, relational, and temporal organization of everyday life. What emerged most clearly from the materials was that uncertainty did not remain where it is often presumed to belong—in clinical encounters, diagnostic processes, or moments of medical explanation. Instead, it moved into the routines through which everyday life had to continue. It shaped how work was inhabited, how competence was conveyed, what could be said or left unsaid, and how a future could still be imagined in the absence of clarity. The four themes that follow trace this movement from the migration of uncertainty beyond the clinic, to the labor of remaining functional under strain, to the management of silence and institutional vulnerability, and finally to the effort to rebuild a livable future.

### Health uncertainty beyond the clinic

Health uncertainty did not remain confined to appointments, tests, or episodes of medical attention. It traveled outward, attaching itself to the structure of ordinary days. One of the clearest features of this experience was that uncertainty began to organize life even when no clinical event was taking place. It was present in mornings, in transitions into work, in the anticipation of ordinary tasks, and in the private calculations required to move through the day without turning uncertainty into a visible public fact. In this sense, uncertainty was not simply something I thought about; it was something I worked around, carried with me, and repeatedly adjusted to as part of everyday functioning.

What made this especially difficult was the absence of a clean boundary between “health” and “the rest of life.” The issue was not that medical concerns occasionally interrupted work. Rather, uncertainty itself became woven into the background conditions under which work was carried out. Teaching, writing, responding, planning, traveling, and maintaining a reliable professional rhythm no longer took place on neutral ground. They were increasingly shaped by internal monitoring of how much energy was available, what could be ignored for now, what had to be postponed, what had to be concealed, and how “normal” the day could still appear from the outside. Under these conditions, work was no longer merely work. It became one of the main places where health uncertainty had to be managed.

One contemporaneous reflection captured the point at which this burden no longer felt divisible into separate problems. I described myself as being in “a complete period of stoppage and inability to act.” In the same reflection, this stoppage did not appear as the result of a single symptom or event, but as the convergence of several pressures, including academic work, family-related distress, financial pressure, institutional conflict and perceived unfairness, conflict at home, and the sense of being repeatedly used by others. The experience was not that one problem had become unbearable in isolation. It was that too many forms of uncertainty had entered the same mental and bodily space at once.

What matters analytically here is not only accumulation, but the disappearance of boundaries. Health uncertainty no longer stood apart from occupational strain, family conflict, institutional injustice, or emotional overload. It entered that whole structure and altered the conditions under which ordinary life could still proceed. In this sense, uncertainty was not an isolated medical concern waiting patiently in the background. It was already participating in the organization of the day.

This movement beyond the clinic changes what kind of problem uncertainty becomes. It is no longer only a question of incomplete knowledge or delayed resolution. It becomes a mode of living in which routine itself is altered. The ordinary is no longer fully ordinary. Tasks that would once have been taken for granted begin to require negotiation, and continuity becomes something that must be actively preserved rather than naturally inhabited. From this perspective, uncertainty functioned less as an interruption and more as an ongoing background condition—one that quietly reorganized the terms under which everyday life could proceed.

The materials also show that this process was cumulative rather than dramatic. There was no single moment in which health uncertainty overtook everything else. Instead, it expanded through repeated noticing, repeated recalibration, repeated effort to continue without clarity, and repeated attempts to prevent private instability from becoming socially visible. Nothing necessarily “stopped,” yet the meaning of continuation changed. Life went on, but not on the same terms.

A later reflection makes that erosion of capacity more explicit:

I could say many things, and I knew that blaming others would not resolve what I was living through. But I had reached a point where I no longer felt physically or mentally able to keep fighting so many battles at once.

Read superficially, this could sound like a statement about exhaustion alone. Analytically, however, it reveals something more consequential, namely the weakening of one’s capacity to continue absorbing uncertainty, conflict, and responsibility without internal cost. This is where biographical disruption becomes especially visible. What is destabilized is not simply health status, but one’s sense of continuity, endurance, and dependable selfhood.

Seen through this lens, the significance of prolonged uncertainty lies in how it unsettled the taken-for-granted structure of everyday life without necessarily producing a single dramatic rupture. Work routines, family responsibilities, and future plans were not abandoned, but they were no longer supported by the same bodily trust or temporal confidence. The result was a form of disruption that often remained invisible to others precisely because life continued outwardly. Uncertainty did not always announce itself through breakdown. More often, it appeared through quiet reorganization.

A further consequence of this was that clinical space lost its monopoly over interpretation. The meaning of uncertainty was not produced only in relation to doctors, tests, or possible diagnoses. It was also produced in offices, classrooms, journeys, family conversations, solitary moments of recalibration, and the practical labor of trying to remain dependable. In that sense, the clinic did not contain the experience; it merely named one part of it. The fuller burden was distributed across the spaces in which ordinary life still had to be sustained.

This helps explain why prolonged health uncertainty should not be understood only as a medical state awaiting clarification. In lived terms, it had already become an occupational, relational, and temporal condition. Before certainty was reached, uncertainty was already at work, reorganizing attention, altering routine, changing the meaning of competence, and reshaping the experience of continuity itself. What emerged in these materials, then, was not simply the story of a health problem extending into life, but the story of life being gradually reorganized around the demands of uncertainty.

### Carrying competence under strain

One of the most demanding aspects of prolonged health uncertainty was not simply fear, confusion, or waiting for clarity. It was the ongoing labor of remaining competent while the internal conditions that once supported competence had begun to shift. Work did not stop. Classes still had to be taught, students still had to be supervised, writing still had to continue, responsibilities still had to be met, and the professional self still had to appear coherent. What changed was the cost of doing these things. Activities that had once been integrated into a familiar rhythm increasingly required calculation, self-monitoring, restraint, and effort. Competence, in other words, could no longer be assumed. It had to be carried.

This distinction matters because visible functioning can easily conceal internal disruption. To others, continuation may appear reassuring. The person is still working, still producing, still meeting obligations, still recognizable as capable, but from within, continuity may be sustained only through invisible labor. In my case, the work of remaining professionally intact involved completing tasks and preserving legibility by continuing to appear reliable, composed, and productive while privately negotiating fatigue, uncertainty, emotional strain, and a changing relationship to my own endurance.

A retrospective self-narrative captured this external continuity in strikingly concrete terms:

Over fifteen years, I kept moving—between campuses, long distances, graduate study, teaching, supervision, publications, and institutional struggle. The numbers themselves were substantial, but what they do not show is how much of that life was built while carrying burdens that rarely became visible to others.

What is important here is not self-congratulation, but compression. Institutional life often recognizes output while concealing the conditions under which output is produced. A curriculum vitae can record movement, productivity, supervision, promotion, and formal achievement, but it cannot show the hidden labor required to preserve those things when ordinary bodily trust and emotional steadiness are under pressure. In that sense, competence was publicly visible as continuity and privately experienced as strain.

A later reflection showed the other side of that same continuity as I wrote, “I have many scientific projects planned, but none of them are moving forward.” This was not simply a statement about lost productivity. It marked the moment when academic work, which had long functioned as one of the few remaining sources of continuity and self-recognition, also began to feel threatened. Competence was therefore not experienced as a secure professional attribute, but as something that had to be repeatedly reassembled under conditions of exhaustion, uncertainty, and strained internal capacity.

This also helps explain why the issue was not reducible to resilience in any celebratory sense. To describe this simply as “coping well” would flatten what the materials actually show. Far from reflecting heroic mastery of uncertainty, functioning had to be repeatedly reconstructed under conditions that made ease impossible. Teaching still happened, but not on neutral terms. Professional responsibility remained, but it had to be met through effortful regulation rather than taken-for-granted flow. The achievement, if it can be called that, was not invulnerability. It was the repeated reassembly of a workable professional self.

A second reflection reveals how closely competence had become tied to conveying rather than receiving support: “I was not asking anyone to carry my burden for me. What I wanted was different. I wanted to keep carrying it myself, but not entirely alone—to have someone walk beside me while I did.” This passage is important because it clarifies the moral structure through which competence was being lived. The struggle was not against dependence alone, nor was it a simple refusal of help. Rather, it reflected an effort to remain responsible without being left entirely alone with that responsibility. What was sought was not rescue, but accompaniment. In occupational terms, this distinction is profound. It shows that competence under strain is not only about endurance; it is also about how much of endurance must be performed in isolation.

Under prolonged uncertainty, this isolation intensified the labor of being “the competent one.” To continue functioning meant carrying one’s tasks along with the expectation of being the person who could continue to carry them. This created a subtle, but powerful trap. The more one remained functional, the easier it became for others—and sometimes for institutions—to read continuation as stability rather than cost. Yet the materials suggest that continued functioning may itself be evidence of hidden expenditure.

Seen through the lens of biographical disruption, this theme shows that disruption does not always present as collapse. It can also appear as over-continuity, expressed through the insistence on staying productive, staying responsible, staying recognizable, even while the internal terms of that recognizability are changing. In such moments, competence no longer describes a stable attribute of the self. It becomes a form of ongoing labor through which professional identity is preserved against erosion.

The significance of this theme, then, lies in making visible the effort required to remain capable when capability itself no longer feels effortless, an effort that institutional life often leaves unexamined. Prolonged health uncertainty did not simply threaten work from the outside. It entered the very conditions under which work was performed. It altered rhythm, cost, and endurance, while leaving the external appearance of functioning largely intact. What emerged from the materials was therefore not a story of uninterrupted strength, but a story of competence maintained through strain and of productivity sustained at a price that remained largely invisible.

### Silence, disclosure, and institutional vulnerability

Uncertainty was not only something I carried internally; it was also something I had to manage socially. What to say, how much to say, when to say it, and to whom—these were never simple matters of honesty or openness. Under prolonged health uncertainty, disclosure did not present itself as a straightforward moral good. It was shaped by risk. To speak was to become potentially legible in ways I could not control. To remain silent was to preserve a degree of protection, but at the cost of carrying more alone. In this sense, silence was not merely absence. It became a strategy of self-management under conditions where visibility did not necessarily promise care.

This was especially significant in institutional life. Professional environments often presume that disclosure will lead to understanding, accommodation, or support. But that presumption depends on trust. Where trust has been weakened by past experiences of unfairness, sanction, or exposure, disclosure can take on a different meaning. It can feel less like relief than like vulnerability. Under such conditions, the question is no longer simply whether one should speak, but whether speaking will be survivable. When I later tried to name what had most deeply damaged my trust, I did not first identify the formal accusation, the punishment, or even the isolation. What remained most vivid was the feeling that unfair power could press down on a person until being right, decent, or hardworking no longer offered protection. I captured that experience with painful clarity in a reflection:

What hurt me most was not the accusation itself, nor the punishment, nor even being left alone. It was the sense that unfair power was trying to crush me—and that being right, being decent, or working hard was not enough to protect me.

This passage reveals something deeper than interpersonal conflict. It speaks to the breakdown of moral predictability, reflected in the loss of confidence that fairness, effort, or professional integrity will be recognized or reciprocated. Once that confidence begins to erode, institutional life can no longer be navigated as a neutral environment. It becomes a site of vigilance. Under such conditions, silence may emerge because disclosing health uncertainty can feel dangerous, even when there is something that needs to be said.

Perceived danger shapes disclosure under health uncertainty. To disclose is to share information while also placing oneself in a field of interpretation shaped by hierarchy, precedent, and institutional memory. In my case, uncertainty was already difficult to carry privately. But the possibility of speaking about it was complicated by a broader experience of institutional vulnerability. The issue involved whether others would understand and whether being more visible would expose me to further misrecognition, weakness, or strategic use by others.

A second reflection makes this institutional injury more explicit:

The most irrational and destructive part was what happened at work. In personal life, conflict can at least be understood as emotional. But in a workplace, responding to wrongdoing should be normal. What became unbearable was seeing those with power use that response against me.

This passage shows that silence and disclosure were health-related questions inseparable from prior experiences of power. The problem extended beyond “Should I tell?” to “What happens to a person once they become more speakable within a setting that has already taught them what visibility can cost?” In this sense, health uncertainty entered an already fragile relation to institutional trust. Silence, then, functioned as a way of managing exposure in an environment where exposure did not feel safe.

The burden of this management was intensified by the need to remain professionally recognizable. I still had to teach, meet responsibilities, and appear coherent. That meant regulating symptoms, fear, and legibility. Too little disclosure risked isolation; too much disclosure risked being re-read through vulnerability. The materials revealed a continuous calibration between openness and concealment, shaping how much of the self could safely appear.

This calibration also helps explain why institutional vulnerability cannot be treated as a secondary issue. It was part of the architecture of uncertainty itself. In practical terms, uncertainty became harder to bear when it had to be carried within settings where justice felt unstable and support could not be presumed. In emotional terms, this created a distinctive form of strain in which “I do not know what is happening” coexisted with “I do not know what it is safe to reveal.” The result was a form of self-containment that was both protective and costly.

A third reflection captures the endpoint of that strain: “No one can carry this much weight alone. Reaching that point does not mean a person has failed; it means they have been left alone with too much, for too long.”

Through the lens of biographical disruption, this theme shows that prolonged uncertainty unsettled continuity in the body and future, as well as continuity in one’s relationship to institutions and social recognizability. The self that must continue functioning is also the self that must calculate safety, credibility, and exposure. Silence, in that context, becomes part of the everyday labor of survival.

The significance of this theme, then, lies in showing that health uncertainty is never lived in an abstract social vacuum. It is interpreted and negotiated within relationships of power. Where institutions feel unreliable, disclosure becomes a risk-laden act rather than a simple resource. The materials revealed silence as a protective response to a world in which visibility could not be trusted, rather than as withdrawal. The cost of that protection, however, included greater isolation, heavier self-containment, and the need to remain outwardly coherent while privately carrying what could not safely be said.

### Rebuilding a livable future

What sustained this experience was the refusal to let uncertainty define the limits of the future entirely, even in the absence of certainty. By the time uncertainty had spread across work, relationships, and selfhood, the question was no longer simply how to endure the present. It had become how to keep the future imaginable. This involved neither imagining a future free of difficulty nor waiting for a moment of total resolution. Rather, it meant trying to rebuild a form of life that still felt livable, one in which work, care, and meaning could continue, even if they could not continue on their previous terms.

This theme is important because coping, in these materials, extended beyond any simple notion of restoration. I was not trying to return to a former version of myself untouched by strain. Nor was I seeking a dramatic rescue. What emerged instead was a quieter, but more demanding, form of future-making centered on remaining responsible, relational, and useful while carrying an altered sense of self and capacity. In that respect, rebuilding a future meant preserving livability in the absence of certainty.

One reflection captures this shift with particular clarity: “I no longer wanted to keep fighting. What I wanted was to be understood—to have someone stand beside me.” This is a crucial turning point. The movement here is from endless struggle to relational recognition, not from weakness to strength. What is being sought is a different social condition under which burden can be carried—not rescue or the removal of burden. Analytically, this matters because it shows that mastery alone was insufficient to make the future imaginable. It became imaginable through the hope that I might no longer have to carry everything in complete emotional isolation.

That desire for accompaniment became even more explicit in another passage: “If she had been beside me, even if nothing else had been fully resolved, that would still have mattered. I only wanted to be seen—to have the fire inside me recognized.” This reveals that the future was not being rebuilt through solutions alone. It was being rebuilt through the possibility of recognition.

To be “seen” here is more than a sentimental wish. It is a condition of survival. It names the difference between merely continuing and continuing in a way that remains humanly bearable. When prolonged uncertainty is carried across work, family life, and institutional strain, being seen becomes part of what makes endurance livable.

This also helps explain why future-making in these materials cannot be reduced to optimism. The materials resist a smooth narrative of growth or recovery. They suggest something more fragile and more credible—a determination not to let exhaustion become the final meaning of the self. This determination remained tied to responsibility. Family life, work, and long-term commitments remained despite intensifying strain. If anything, they made withdrawal harder. The future therefore had to be rebuilt within and through ongoing obligations.

A later reflection made this responsibility more concrete in relation to family and inheritance. As I tried to understand what my health condition might mean for my children and future generations, uncertainty persisted in a different form. It became something to be documented, explained, and carried forward as usable knowledge rather than only fear. In that moment, I wrote that “we may have a chance to end a disease in one family.” This left the uncertainty surrounding my own body unresolved while altering the moral horizon of the experience. The future became imaginable through the possibility of leaving others better prepared than I had been, even without a return to safety.

Another later reflection expressed this orientation especially directly when I wrote, “I have no desire for revenge. I only want to become strong again—and to be of help to others.”

Taken together, these reflections show that the desired future was oriented toward preparation, contribution, and moral re-grounding rather than vindication, domination, or personal repair alone. Becoming strong again meant something other than returning to invulnerability. It was tied to the desire to do good, to remain generative, and to recover a self that could still act meaningfully in the world. In this sense, future-making was also ethical work.

Seen through the lens of biographical disruption, this theme shows that disruption unsettles continuity and forces the question of what kind of future can still be inhabited. Under prolonged uncertainty, the future cannot be taken for granted. It must be reimagined under altered conditions. The task, then, is to build around vulnerability without trying to erase it and to find forms of life, relationship, and work that can remain possible even when certainty does not return on demand.

The significance of this theme lies in showing that prolonged uncertainty need not end in closure. In the materials examined here, what emerged instead was a struggle to preserve livability by remaining able to work, to care, to be recognized, and to move forward without surrendering either responsibility or dignity. The future, in that sense, was reimagined as a practical and moral project—fragile, incomplete, but still worth inhabiting—without becoming a stable promise.

## Discussion

This article has examined how prolonged health uncertainty entered work, family life, and selfhood as a lived condition that extended beyond a discrete medical episode and gradually reorganized everyday continuity. Across the four themes, the analysis suggests that uncertainty was experienced less as a temporary gap in knowledge than as an ongoing condition of occupational, relational, and emotional management. What began as a health-related uncertainty extended beyond the clinic; it spread into routines, altered the cost of competence, complicated decisions about disclosure, and reshaped what kind of future could still be imagined. In that sense, this autoethnography contributes to health-oriented qualitative scholarship by showing that uncertainty may become biographically disruptive even before clinical clarity is achieved.

The concept of biographical disruption (Bury, 1982) provides the primary lens for interpreting the patterns traced here. However, this article extends that concept in an important way. In classic formulations, disruption is often associated with the onset or recognition of chronic illness as a rupturing event in the life course. Here, disruption emerged cumulatively through prolonged ambiguity rather than from a single dramatic diagnostic moment. Ordinary continuity was gradually reworked through recalibration, self-monitoring, emotional containment, and repeated efforts to preserve functionality in the absence of certainty. The analysis therefore suggests that uncertainty itself—not only diagnosis or illness identity—can operate as a disruptive force, reorganizing time, meaning, and self-continuity long before resolution is reached.

The analysis also resonates with scholarship on illness work and the ongoing management of chronic conditions in everyday life (Corbin and Strauss, 1985, Corbin and Strauss, 1988). Yet what this autoethnography makes especially visible is the hidden labor required to remain “apparently functional” while internally reorganizing around uncertainty. Work continued, responsibilities remained, and competence was often still legible to others, but the internal conditions that supported that competence had shifted. This is an important point because health-related disruption is often socially recognized only when visible breakdown occurs. The materials suggest that continued performance may itself conceal substantial strain. The labor of functioning—teaching, producing, caring, responding, remaining coherent—persisted under uncertainty, becoming effortful, costly, and increasingly dependent on invisible forms of self-regulation. In this respect, this article aligns with narrative and interpretive work showing that illness-related experience is endured and continually reorganized through meaning-making and adaptation (Charmaz, 1991, Frank, 1995, Hydén, 1997).

A further contribution of this autoethnography lies in its attention to silence, disclosure, and institutional vulnerability. Research on illness uncertainty has often focused on patient interpretation, communication, and coping (Mishel, 1988, Mishel, 1990), while chronic illness scholarship has more broadly emphasized identity, adaptation, and daily management. What emerged here, however, was that disclosure was more than a simple matter of openness or help-seeking. It was shaped by prior experiences of unfairness, exposure, and institutional distrust. Under those conditions, silence went beyond withholding and functioned as a protective form of self-management. This matters because it locates health uncertainty within relations of power rather than within the self alone. The question concerned both “What is happening to me?” and “What happens if this becomes more visible?” Seen this way, uncertainty is not only epistemic or emotional; it is also social and organizational, shaped by whether one’s environment is perceived as trustworthy enough to bear disclosure without converting vulnerability into risk. From an occupational perspective, attention must extend beyond whether a person remains at work to the invisible labor, energy trade-offs, and disclosure calculations required to remain there (Tomas et al., 2022).

The final contribution concerns future-making. My account suggests that coping under prolonged uncertainty was not adequately described as optimism, resilience, or recovery in any uncomplicated sense. What was being rebuilt was livability in the absence of certainty. The effort was to preserve a future that could still be inhabited morally, relationally, and practically. This involved remaining responsible to others, sustaining a professional identity, and seeking recognition and accompaniment rather than rescue. Here, the analysis adds to the literature on illness narratives by showing that future reconstruction may take place without narrative closure, through ongoing efforts to make vulnerability compatible with work, care, and dignity (Frank, 1995). The future, in this account, was rebuilt as a fragile project of continuation without becoming a stable promise.

It is also important to acknowledge that the materials could support more than one analytic emphasis. They might be read affirmatively as evidence of endurance, resilience, and an academic life sustained despite strain. They might also be read more critically as evidence of over-functioning, in which competence continued to be performed at considerable private cost. The interpretation offered here foregrounds livability rather than recovery, breakdown, or resilience alone because this emphasis remained closest to the language and tensions of the materials themselves. In this sense, the analysis remains open to alternative readings while offering one situated interpretation of how uncertainty, work, responsibility, and future-making became linked.

Methodologically, analytic autoethnography makes visible forms of burden that are difficult to capture through externally oriented accounts alone (Anderson, 2006). Prolonged health uncertainty often leaves few visible events dramatic enough to anchor conventional analysis, even while quietly reshaping the conditions of everyday life. Autoethnography makes it possible to examine those slow reorganizations from within, while still treating them as analytically meaningful rather than merely personal. At the same time, this autoethnography remains limited by its focus on a single life and a single situated archive. Its aim is analytic insight rather than statistical generalization. What it offers is a close account of how health uncertainty can become embedded in work, family life, and selfhood in ways that may resonate with, rather than represent, others’ experiences.

Taken together, the four themes argue for understanding prolonged health uncertainty as more than a private medical problem awaiting diagnosis. It can become a disruptive occupational and relational condition in its own right. By showing how uncertainty was carried across routine, competence, institutional life, and future-making, the analysis brings into view a form of burden that may remain hidden precisely because outward continuity is preserved. What appears, from the outside, as ongoing functioning may in fact be the product of sustained and costly labor. Making that hidden labor visible is one of the central contributions of this autoethnography.

## Conclusions

This autoethnography has argued that prolonged health uncertainty should be understood as more than a medical problem awaiting clarification. As the analysis showed, uncertainty may already be reshaping everyday life long before diagnostic resolution is reached. Here, uncertainty moved beyond the clinic and became embedded in work routines, family responsibilities, emotional containment, and the ongoing effort to preserve a coherent sense of self. The disruption reached beyond bodily confidence to continuity itself, understood as the taken-for-granted ability to move through work, care, and future planning without having to renegotiate them at every turn.

By foregrounding this lived process, this autoethnography contributes to qualitative health scholarship in two ways. First, it shows that biographical disruption may emerge through illness as a recognized event or through prolonged uncertainty as an accumulating condition. Second, it makes visible the hidden labor involved in remaining “apparently functional.” Continued work and outward competence could coexist with disruption; they often depended on self-monitoring, selective silence, emotional regulation, and repeated efforts to preserve continuity under strain. What appears from the outside as ongoing normality may therefore conceal substantial biographical and occupational cost.

These insights also have broader practical relevance. They suggest that health-related uncertainty can affect working life through visible incapacity as well as through invisible effort, altered endurance, and the need to manage disclosure within uneven relations of trust. From this perspective, whether a person remains at work is only part of the issue; the hidden labor, energy trade-offs, and relational calculations required to remain there also matter. This matters for how institutions, colleagues, and workplaces understand functioning, support, and vulnerability. It also matters for occupational health thinking more broadly, because the burden of uncertainty may remain unrecognized precisely when outward performance is preserved.

At the same time, the materials suggest that coping under prolonged uncertainty resists simplified narratives of resilience or recovery. What emerged here was a more fragile and demanding effort to preserve livability. That effort involved endurance, recognition, accompaniment, and the rebuilding of a future that could still be inhabited with responsibility and dignity. The future was reconstructed through continuation without the restoration of certainty.

As an analytic autoethnography, this story makes no claim to representativeness. Its contribution is interpretive rather than generalizable in a statistical sense. Yet that is precisely where its value lies. By tracing how uncertainty was lived from within, across work, family life, and selfhood, it brings into view a form of burden that may otherwise remain hidden beneath the appearance of ordinary functioning. Prolonged health uncertainty reorganizes life while answers remain unavailable. To understand that reorganization is to recognize that uncertainty is a matter of diagnosis as well as work, relationship, endurance, and the fragile labor of remaining intact.

## CRediT authorship contribution statement

**Mehmed Zahid Çögenli:** Writing – review & editing, Writing – original draft, Methodology, Investigation, Formal analysis, Data curation, Conceptualization.

## Declaration of Generative AI and AI-assisted technologies in the writing process

During the preparation of this manuscript, the author used ChatGPT solely to assist with language editing. The author takes full responsibility for the final content of the manuscript.

## Ethics approval and consent to participate

Not applicable. This study draws exclusively on the author’s own self-authored materials and did not involve the recruitment of external participants.

## Funding

This research did not receive any specific grant from funding agencies in the public, commercial, or not-for-profit sectors.

## Declaration of Competing Interests

The author declare that they have no known competing financial interests or personal relationships that could have appeared to influence the work reported in this paper.

## Data Availability

The materials underlying this article are not publicly available because they consist of self-authored documents containing sensitive personal, relational, and potentially identifying information. Selected de-identified excerpts are included in the article. Additional materials are not available in order to protect privacy and confidentiality.
